# RAPTOR promotes colorectal cancer proliferation by inducing mTORC1 and upregulating ribosome assembly factor URB1

**DOI:** 10.1002/cam4.2810

**Published:** 2019-12-30

**Authors:** Tao Wang, Wei‐Sheng Zhang, Zheng‐Xia Wang, Zhi‐Wei Wu, Bin‐Bin Du, Lai‐Yuan Li, Yi‐Feng Chen, Xiong‐Fei Yang, Xiang‐Yong Hao, Tian‐Kang Guo

**Affiliations:** ^1^ The First School of Clinical Medicine Lanzhou University Lanzhou China; ^2^ Department of Colorectal Surgery Gansu Provincial People's Hospital Lanzhou China; ^3^ Department of Otolaryngology The Second Hospital of Lanzhou University Lanzhou China; ^4^ The School of Preclinical Medicine Gansu University of Chinese Medicine Lanzhou China; ^5^ Department of General surgery Gansu Provincial People's Hospital Lanzhou China

**Keywords:** colorectal cancer, cyclinA2, mTORC1, proliferation, RAPTOR, URB1

## Abstract

Mammalian target of rapamycin complex 1 (mTORC1) is evolutionally conserved and frequently activated in various tumors, including colorectal cancer (CRC). It has been reported that the ribosome assembly factor Urb1 acts downstream of mTORC1/raptor signaling and contributes to digestive organ development in zebrafish. Previously, we highlighted that URB1 was overexpressed in CRC. Here, we assessed the mTORC1/regulatory associated protein with mTOR (RAPTOR)‐URB1 axis in CRC tumorigenesis. We found that RAPTOR was overexpressed in CRC tissues and cell lines, was a favorable predictor in patients with CRC, and positively correlated with URB1. Silencing of RAPTOR suppressed CRC cell proliferation and migration and induced cell cycle arrest and apoptosis in vitro and inhibited xenograft growth in vivo. Moreover, ectopic overexpression of RAPTOR exerted an inverse biological phenotype. Knockdown of RAPTOR quenched mTORC1 activity and reduced the expression of URB1 and cyclinA2 (CCNA2). In contrast, overexpression of RAPTOR activated mTORC1 and upregulated URB1 and CCNA2. Furthermore, URB1 and CCNA2 expression were also impeded by rapamycin, which is a specific inhibitor of mTORC1. Thus, RAPTOR promoted CRC proliferation, migration, and cell cycle progression by inducing mTORC1 signaling and transcriptional activation of both URB1 and CCNA2. Taken together, we concluded that RAPTOR has the potential to serve as a novel biomarker and therapeutic target for CRC.

## Graphical abstract

Regulatory associated protein with mammalian target of rapamycin (RAPTOR) contributes to colorectal cancer proliferation and cell cycle progression through positively regulating URB1 and cyclinA2 via activating mTOR complex 1 signaling. We conclude that RAPTOR has the potential to serve as a novel biomarker and therapeutic target for colorectal cancer.

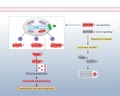

## 1 INTRODUCTION

Mammalian/mechanistic target of rapamycin (mTOR), an evolutionarily conserved serine/threonine protein kinase in the PI3K‐related kinase family, plays a critical role in cellular processes related to tumorigenesis, metabolism, immune function, and aging.^1^ Mammalian/mechanistic target of rapamycin is the catalytic core of two signaling complexes, mTOR complex 1 (mTORC1) and complex 2 (mTORC2). Mammalian target of rapamycin complex 1, an evolutionarily conserved multiprotein complex, is specifically sensitive to rapamycin and functions as a key regulator of cell growth by promoting gene transcription, protein translation, ribosome biogenesis, and autophagy.^2^, ^3^ Increasing reports have shown that mTORC1 is frequently activated in various cancers, particularly in digestive malignant tumors, such as colorectal cancer (CRC),^4^ gastric cancer,^5^ pancreatic ductal adenocarcinoma (PDAC),^6^ and hepatocellular carcinoma (HCC).^7^ Although the activity of mTORC1 can be counteracted by the clinical inhibitor, everolimus, there still exists a formidable obstacle of extensive clinical application due to resistance resulting from the limitation of everolimus on the mTORC1 cascade and complex tumor microenvironment.^8^, ^9^, ^10^ Therefore, exploring mTORC1 signaling is an urgent priority since its exploitation may remedy the therapeutic limitations of rapamycin analogues.

The following three core components define mTORC1: (a) mTOR; (b) regulatory associated protein with mTOR (RAPTOR); and (c) mammalian lethal with Sec13 protein 8.^2^ Regulatory associated protein with mTOR is the scaffold protein and facilitates substrate recruitment to mTORC1 through directly binding to the TOR signaling motif. Regulatory associated protein with mTOR is also essential for the lysosomal surface localization of mTORC1, which activates mTORC1 and propels its pro‐proliferation function.^3^, ^11^ Intriguingly, several studies have illustrated that RAPTOR is responsible for the induction of mTORC1 during tumorigenesis and progression. The interaction of RAPTOR and SHOC2 has been shown to inhibit RAPTOR‐mTOR binding and induce autophagy via inactivating mTORC1, which facilitates, in parallel, RAS‐ERK activation and PDAC progression.^12^ Autophagy induced by cardamonin is associated with mTORC1 inhibition through decreasing RAPTOR in cervical cancer.^13^ Moreover, RAPTOR upregulation has been shown to contribute to PI3K‐mTOR inhibitor resistance in renal cancer,^14^ further indicating the effect of RAPTOR on mTORC1 during tumor progression and outcomes. Therefore, RAPTOR might be a favorable molecule to target in order to solve the issue of mTORC1‐targeted preparations in tumor treatment. However, the downstream target of mTORC1/RAPTOR signaling remains to be further elucidated.

Given that mTORC1 positively regulates several steps in ribosome biogenesis, including synthesis of ribosome proteins and components required for ribosome assembly,^15^ and that ribosome assembly factors are mostly conserved evolutionarily,^16^, ^17^, ^18^ it is extremely attractive to investigate the reciprocity of mTORC1/RAPTOR signaling and ribosome assembly factors. URB1, the human homolog of Npa1 in *Saccharomyces cerevisiae*, is a classical ribosome assembly factor required for the 60S ribosome subunit assembly.^19^ An increasing number of studies have uncovered that URB1 plays a vital role in cell proliferation and organogenesis. URB1 frameshift mutations result in a complete loss‐of‐function, which, in turn, leads to embryonic lethality caused by truncated protein synthesis and hypoplasia.^20^ Urb1 also plays a pivotal role in governing ribosome biogenesis and cell growth downstream of mTORC1/raptor signaling, in addition to regulating the development of digestive organs in zebrafish,^21^ which enhances the implication of URB1 and RAPTOR coaction in glandular epithelial cell proliferation.

In an ongoing study, we have validated that the ribosomal assembly factor, URB1, promotes CRC proliferation and upregulates cyclinA2 (CCNA2). Thus, we speculated that mTORC1/RAPTOR signaling may promote digestive malignant tumor proliferation and progression by activating the novel oncogene, URB1. Here, we provided evidence that RAPTOR and URB1 were collectively overexpressed in CRC, and that URB1 expression was positively associated with RAPTOR. Silencing of RAPTOR inhibited CRC cell proliferation and migration via inactivating mTORC1 and downregulating URB1 and CCNA2. Combined with its prognostic significance, RAPTOR may serve as a potential biomarker and therapeutic target for CRC.

## 2 MATERIALS AND METHODS

### 2.1 CRC clinical specimens and tissue microarray

A total of 40 paired cancer tissues and adjacent normal tissues were obtained from patients with CRC who underwent surgical resection in Gansu Provincial People's Hospital (Lanzhou, China) from April‐August 2018. None of these patients were pretreated with chemotherapy or radiotherapy prior to surgery. The adjacent normal tissues were not less than 5 cm away from the cranial incisal margin of cancer. All specimens were confirmed to be normal or cancerous tissue via histopathological methods. All tissues were used with written informed consent from the patients, and this study was approved by the Ethics Committee of Gansu Provincial People's Hospital. Tissue microarray (TMA) slides containing 101 pairs of CRC tissue samples were purchased from Shanghai Outdo Biotech Co., Ltd. Clinical specimens and TMA slides were processed using routine methods for quantitative PCR or immunohistochemistry (IHC). Clinicopathological characteristics, including gender, age, TNM stage, histological grade, pathological subtype, and overall survival (OS) were acquired and analyzed based on the medical records of TMA.

### 2.2 Immunohistochemistry

Consecutive sections were deparaffinized in xylene, rehydrated in an alcohol gradient, and submerged in EDTA as an antigenic retrieval buffer. Endogenous peroxidase activity was blocked by incubation in 3% H_2_O_2_. The arrays were incubated overnight at 4°C with indicated antibodies. Slides were developed with diaminobenzidine and counterstained with hematoxylin. Specific antibodies against RAPTOR (1:100; Abcam), URB1 (1:100; Abcam), CCNA2 (1:500; Abcam), Ki67 (1:200; Abcam), phosphor‐p70S6K (S418) (1:200; Immunoway), and phospho‐4EBP1 (S65) (1:200; Immunoway) were used. When analyzing the level of protein expression, negative was defined as no or weak staining in cells, whereas positive expression was defined as distinct or strong staining in more than 20% cells.^22^ High or low expression level of RAPTOR was assessed as previously described.^17^ The relationship between RAPTOR expression and clinicopathological characteristics was analyzed using a Chi‐square test.

### 2.3 Cell culture and chemical inhibitor

The normal human colon epithelial cell line NCM460 and human CRC cell lines (LoVo, RKO, HCT116, and SW480) were obtained from the Cell Band of the Chinese Academy of Science (Shanghai, China). LoVo, RKO, and SW480 cells were maintained in high glucose DMEM medium (Gibco), and HCT116 cells were cultured in McCoy's 5A medium (Sciencell). Both mediums were supplemented with 10% fetal bovine serum (FBS; Thermo Fisher Scientific) and 1% penicillin/streptomycin in a humid incubator with 5% CO_2_ at 37°C. The chemical inhibitor, rapamycin (MedChemExpress), was used to treat cells at concentrations of 50 and 100 nmol/L.

### 2.4 Quantitative real‐time PCR

Total RNA from tissue samples or cultured cells was isolated using TRIzol reagent (Sigma‐Aldrich) according to the manufacturer's instructions. RNA was reverse transcribed using PrimeScript RT reagent Kit (Takara Japan). The relative levels of *RAPTOR*, *URB1*, *CCNA2*, *mTOR*, *4EBP1*, *p70S6K*, and *glyceraldehyde 3-phosphate dehydrogenase* (*GAPDH*) mRNA were evaluated by quantitative real‐time PCR (qRT‐PCR) using SYBR Master Mixture (Takara Bio). Expression of *GAPDH* mRNA served as an internal control for normalization.

### 2.5 Western blotting

The cells were collected and resuspended in lysis buffer (RIPA, KeyGEN). Then, the cell lysates were centrifuged and the supernatants were collected. Total protein was extracted using RIPA lysis buffer (Thermo Fisher Scientific), resolved by sodium dodecyl sulfate‐polyacrylamide gel electrophoresis, electrotransferred to polyvinylidene fluoride membranes, and incubated overnight with primary antibodies as follows: RAPTOR (1:1000; Abcam), URB1 (1:500; Abcam), CCNA2 (1:500; Abcam), mTOR (1:2000; Immunoway), phosphorylated (phospho)‐mTOR (Ser2448) (1:2000; Immunoway), 4EBP1 (1:2000; Immunoway), phospho‐4EBP1 (Ser65) (1:1500; Immunoway), p70S6K (1:1000; Immunoway), phospho‐p70S6K (Ser418) (1:1500; Immunoway), RPS6 (1:1000; Abcam), phosphor‐RPS6 (Ser235 + Ser236) (1:1000; Abcam) and GAPDH (1:2500; Abcam). GAPDH was used as a loading control.

### 2.6 Lentivirus and transfection

An overexpression sequence, two short hairpin RNAs (shRNAs) of *RAPTOR*, and a triple‐plasmid system (pSPAX2, pMD2G, and a shuttle plasmid that carried the target gene or shRNA) were all purchased from Hanbio Biotech Co., Ltd. The overexpression sequence or shRNAs were cloned into vectors in order to construct lentiviral vector‐based overexpression or interference based on these target sequences, respectively. The lentiviral vectors encoding overexpression of *RAPTOR* (RAPTOR), control vector (Vector), or shRNAs of *RAPTOR* (sh1, sh2), or nonsense control sequence (nc) were added into cultured cells according to the instructions recommended by the manufacturer. Transfection efficiency was evaluated by qRT‐PCR and western blot.

The sequences used are as follows: *RAPTOR‐F*: AGAGGATCTATTTCCGGTGAATTCGCCACCATGGAGTCCGAAATGCTGC; *RAPTOR‐R*: CACTTAAGCTTGGTACCGAGGATCCTCTGACACGCTTCTCCACCGAGTA; sh1: 5′‐GATCCGTCTGGAAACCATCGGTGCAAATTTATTCAAGAGATAAATTTGCACCGATGGTTTCCAGATTTTTT‐3′; sh2: 5′‐GATCCGCGGAAAGGATTATGAGGTCGTATATTCAAGAGATATACGACCTCATAATCCTTTCCGCTTTTTT‐3′; nc: 5′‐GATCCGTTCTCCGAACGTGTCACGTAATTCAAGAGATTACGTGACACGTTCGGAGAATTTTTT‐3′.

### 2.7 Proliferation assay

A Cell Counting Kit‐8 assay (Wanlei Bio) was used to measure cell proliferation. The viability of CRC cells (5 × 10^4^ cells per well in 96‐well plates) was monitored at 0 and 48 hours after transfection following the manufacturer's instructions. The absorbance was detected at OD490 nm by a microplate reader (Multiskan^TM^ GO; Thermo Fisher Scientific).

### 2.8 Colony formation

A total of 200 cells were seeded into a 6‐well plate and cultured in medium containing 10% FBS. The cells were incubated for 24 hours at 37°C with 5% CO_2_. The medium was changed every 3 days, and the cells were cultured until the termination of colony formation. The cells were washed with phosphate buffer saline (PBS), fixed with triformol, and then stained with crystal violet (0.1%) for 10‐20 minutes. Visible colonies were manually counted.

### 2.9 Migration assay

A 6‐well plate was divided into six parts by drawing five lines with equal lateral distributions across each well using a disinfectant marker pen prior to seeding 5 × 10^5^ cells. On the second day, the confluent cells was scratched with a pipette tip held perpendicular to the five lateral lines. Cell debris was removed by washing once with PBS. After adding medium containing 1% serum, the cells were incubated at 37°C with 5% CO_2_ and monitored at 0, 24, and 48 hours. The migration distance was calculated using ImageJ software (Rawak Software).

### 2.10 Cell cycle and apoptosis assay

Logarithmic growth cells were trypsinized, seeded into a 6‐well plate at a density of 5 × 10^5^ cells per well, and then incubated at 5% CO_2_, at 37°C for 48 hours. The cells were then centrifuged, washed twice in PBS, and incubated with 100 μL of RNase A at 37°C for 30 minutes. Afterwards, the cells were resuspended in 400 μL of propidium iodide and incubated at 4°C in the dark for 30 minutes. Finally, flow cytometry (FCM) was employed to examine the cell cycle distribution and apoptosis. The excitation wavelength was 488 and 535 nm.

### 2.11 Tumorigenesis in nude mice

The in vivo experiments performed in this study were approved by the Ethics Committee of Gansu Provincial People's Hospital. Twenty female BALB/c nude mice (4 weeks old), purchased from SLCA Laboratory Animal Co., Ltd., were used to investigate xenograft formation of human CRC cells. RKO cells transfected with RAPTOR shRNA (sh‐RAPTOR) or ctrl shRNA (sh‐NC) and HCT116 cells transfected with the control vector (Vector) or RAPTOR overexpression vector (RAPTOR) were injected into the right axilla subcutaneous of nude mice. The weight of the mice and volume of xenografts were calculated every seven days starting on day 7 after injection. After four weeks, all of the mice were sacrificed with 5% carbon monoxide, and then the xenograft tissues were excised and fixed with 10% formalin for further study.

### 2.12 Statistical analysis

The continuous variables are presented as the mean ± SD. An independent sample *t* test was used to assess significant differences between two groups, and differences among three or more groups were compared using one‐way ANOVA. Pearson correlation analysis was conducted to evaluate the relevance between RAPTOR and URB1 expression. The *χ* ^2^ test and Kaplan‐Meier survival analysis were employed to analyze the associations of RAPTOR expression level with clinicopathological parameters and OS *P* < .05 was defined as statistically significant. We made use of GraphPad Prism 7.0 software and SPSS 20.0 (IBM) to calculate all statistics.

## 3 RESULTS

### 3.1 Overexpression of RAPTOR in human CRC is positively related to URB1 expression and correlates with vicious clinicopathological features and infaust prognosis

**Figure 1:**
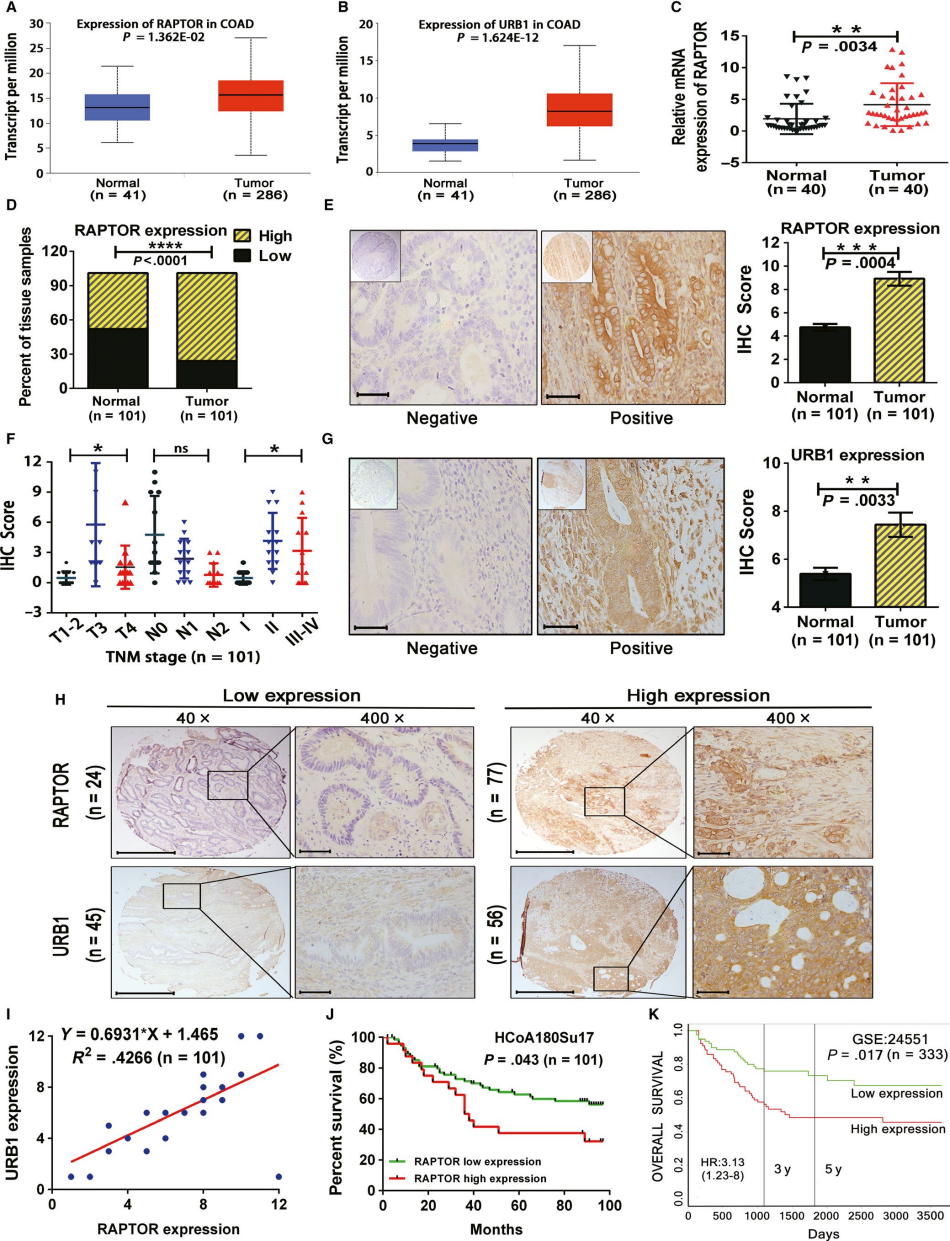
Regulatory associated protein with mammalian/mechanistic target of rapamycin (RAPTOR) is overexpressed in colorectal cancer (CRC) tissues and is positively associated with URB1 expression, poor clinicopathological features, and prognosis of patients with CRC. A and B, Expression of RAPTOR and URB1 in human colon cancer tissues based on The Cancer Genome Atlas (TCGA) datasets (http://ualcan.path.uab.edu). C, RAPTOR mRNA expression level was measured in paired clinical CRC samples by using quantitative real‐time PCR. D and E, RAPTOR protein expression level was assessed in clinical CRC tissue microarray (TMA) through immunohistochemistry (IHC) staining. Scale bars: 50 μm (×400 magnification). F, Overexpression of RAPTOR was associated with a greater T stage and TNM stage in patients with CRC. G, URB1 protein expression level was assessed in TMA through IHC staining. Scale bars: 50 μm (×400 magnification). H and I, RAPTOR exhibited a synchronized expression pattern and was positively correlated with URB1 by utilizing IHC analysis. Scale bars: 100 μm (×40 magnification). Scale bars: 50 μm (×400 magnification). J and K, Patients with CRC had a high expression of RAPTOR and showed inferior overall survival as compared to that of patients with a low expression of RAPTOR based on this study and Gene Expression Omnibus data. Data are mean ± SD, ns, not significant, *P < .05, **P < .01, ***P < .001 and ****P < .0001

By visiting The Cancer Genome Atlas (TCGA) database (http://ualcan.path.uab.edu), we preferentially consulted RAPTOR and URB1 expression in colon cancer and found that the two are elevated in tumor tissue as compared to in para‐tumor tissue (Figure 1A,B). We then examined RAPTOR mRNA expression level in 40 paired CRC samples and found that the tumor tissues had higher RAPTOR expression in comparison to that of normal tissues (*P* = .0034; Figure 1C). To validate the relationship between RAPTOR expression and human CRC, TMA slides originating from 101 CRC patients were assessed using IHC staining. Consistent with TCGA data, RAPTOR was highly expressed in 76.24% (77/101) colon cancer specimens, whereas its high expression was detected only in 48.51% (49/101) of paired adjacent normal samples (*P* < .0001; Figure 1D,E). Furthermore, clinicopathological analysis revealed that RAPTOR overexpression was intimately correlated with the T stage and TNM stage (Figure 1F), but not with the other clinicopathological characteristics (Table 1).

**Table 1:** Correlation between RAPTOR expression and clinicopathologic characteristics

Characteristics	No. of cases	RAPTOR expression		*P* value^a^
		Low	High	
Age (y)				
≤60	22	5	17	.897
>60	79	19	60	
Gender				
Male	50	15	35	.145
Female	51	9	42	
Pathologic subtype				
Adenocarcinoma	63	14	49	.640
Mucinous adenocarcinoma	38	10	28	
Pathological grading				
pG1	23	5	18	.965
pG2	62	15	47	
pG3	16	4	12	
Lymph node status				
N0	61	12	49	.340
N1	30	8	22	
N2	10	4	6	
Primary tumor				
T1, T2	6	4	2	.0224*
T3	75	14	61	
T4	20	6	14	
TNM stage				
I	6	1	5	.0143*
II	54	19	35	
III‐IV	41	4	37	

Abbreviation: RAPTOR, regulatory associated protein with mTOR.

a *χ* ^2^ test.

* Significant correlation.

Given that URB1 is also overexpressed in colon cancer tissues according to TCGA dataset, we then assessed URB1 expression in TMA slides, and found that URB1 protein was expressed significantly higher in colon cancer tissues than normal tissues (*P* = .0033; Figure 1G). Additionally, correlation analysis demonstrated that URB1 was positively related to RAPTOR expression (Figure 1H,I). In addition, Kaplan‐Meier analysis revealed that colon cancer with higher RAPTOR expression had a poorer OS (*P* = .043; Figure 1J), which was compatible with the GEO database (Figure 1K) (http://genomics.jefferson.edu/proggene). These results indicated that overexpression of RAPTOR correlated with a more advanced TNM stage and poorer OS, implying that RAPTOR might be a potential biomarker for patients with CRC.

### 3.2 RAPTOR silencing suppresses the proliferation and colony formation of CRC cells

**Figure 2:**
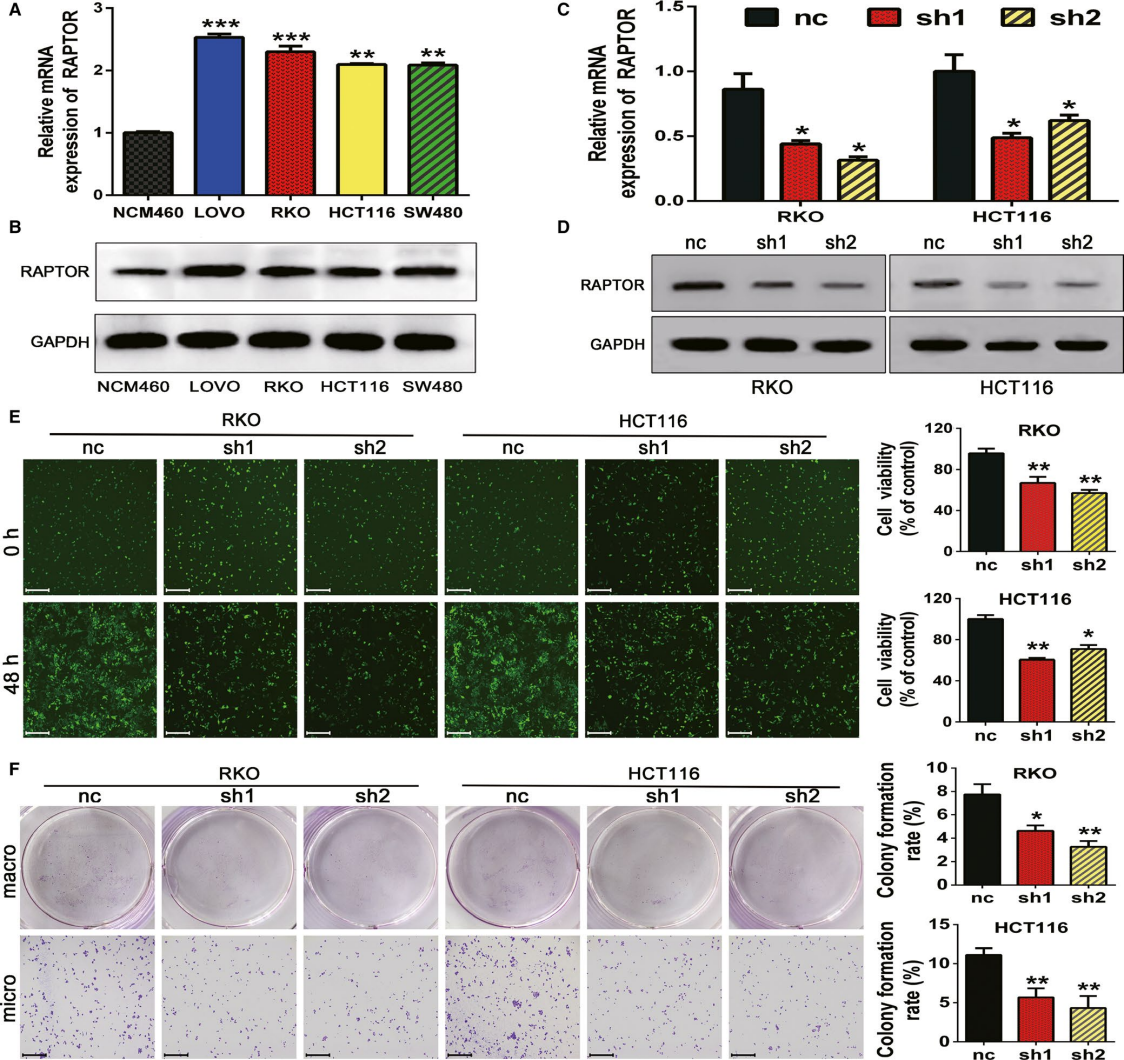
Regulatory associated protein with mammalian/mechanistic target of rapamycin (RAPTOR) silencing inhibits the proliferation and colony formation of colorectal cancer (CRC) cells. A and B, RAPTOR mRNA expression in a normal human colon epithelial cell line (NCM460) and human CRC cell lines (RKO, SW480, HCT116, and LoVo) was measured using quantitative real‐time PCR and western blot. C and D, The mRNA and protein expression of RAPTOR in RKO and HCT116 cells were reduced by two specific shRNA as assessed by qRTPCR and Western blotting, respectively. E and F, The cell viability and colony formation of RKO and HCT116 were suppressed by two shRNAs 48 h post‐transfection. The cell counts were calculated by a Cell Counting Kit‐8 assay. Scale bars: 50 μm (×100 magnification). Data are mean ± SD (n = 3), *P < .05, **P < .01 and ***P < .001

We examined endogenous mRNA and protein expression of RAPTOR in several CRC cell lines—including RKO, SW480, HCT116, and LoVo—and the normal colon epithelial cell line, NCM460, by qRT‐PCR and Western blotting. Four CRC cell lines exhibited relatively high expression levels of RAPTOR, among which the RKO and HCT116 cell lines were selected to be used for lentivirus transfection assays (Figure 2A,B). Our results showed that two shRNAs efficiently decreased RAPTOR mRNA and protein expression in RKO and HCT116 cells (Figure 2C,D). Furthermore, we confirmed the proliferative function of RAPTOR on CRC cells. CCK8 and colony formation assays revealed that RAPTOR silencing markedly suppressed the proliferation and colony formation of CRC cells (Figure 2E,F), which indicated that RAPTOR might play an oncogenic role in CRC tumorigenesis.

### 3.3 Inhibition of RAPTOR induces cell cycle arrest and apoptosis and hampers migration in CRC cells

**Figure 3:**
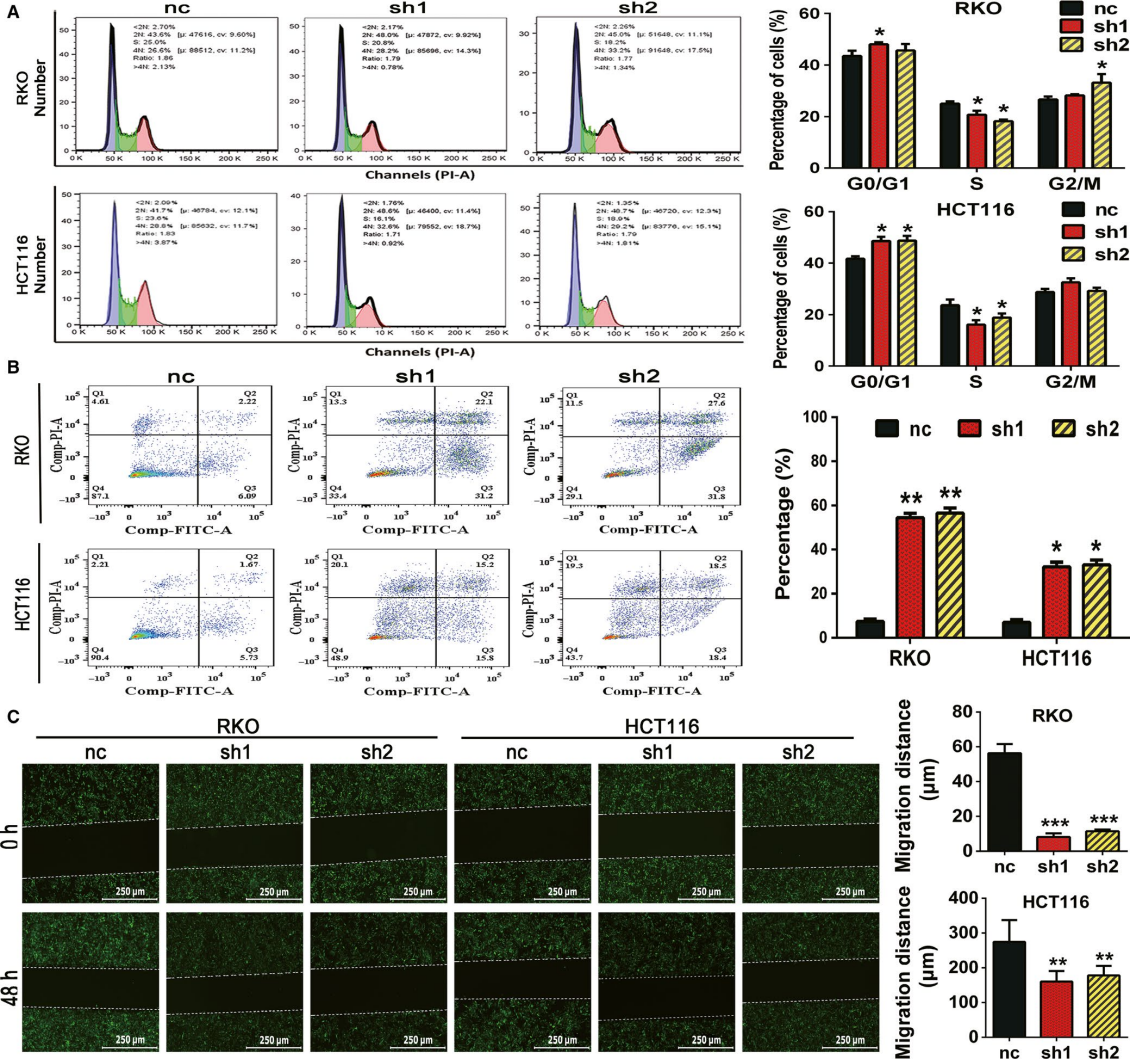
Silencing of regulatory associated protein with mammalian/mechanistic target of rapamycin (RAPTOR) induces cell cycle arrest and apoptosis and inhibits migration in colorectal cancer (CRC) cells. A, HCT116 cells and some RKO cells were arrested in the G0/G1 phase and were decreased in the S phase by RAPTOR knockdown. B, The effect of RAPTOR silencing on cell apoptosis was measured by using flow cytometry analysis. C, Migration of the indicated cells was evaluated by using a wound healing assay. Representative images were taken at 0 and 48 h after scratching. Scale bars: 250 μm (×100 magnification). Data are mean ± SD (n = 3), *P < .05, **P < .01 and ***P < .001

We used a FCM assay to determinate the role of RAPTOR on cell cycle transition and apoptosis. According to the images and analysis, cellular inhibition of RAPTOR induced cell cycle arrest and apoptosis in CRC cells (Figure 3A,B). We then evaluated the effect of RAPTOR on the migration behavior of CRC cells. The migration capability was monitored, and pictures were collected at 0 and 48 hours posttransfection of the shRNA lentivirus. The micrographs showed a significantly shorter average migration distance in RAPTOR‐silenced cells as compared to the control groups (Figure 3C). These results suggested that deletion of RAPTOR blocks cell‐cycle transition, induces apoptosis, and simultaneously inhibits the migration ability of CRC cells.

### 3.4 RAPTOR silencing deactivates mTORC1 signaling and exhibits a similar effect to rapamycin in downregulating URB1 and CCNA2 expression

**Figure 4:**
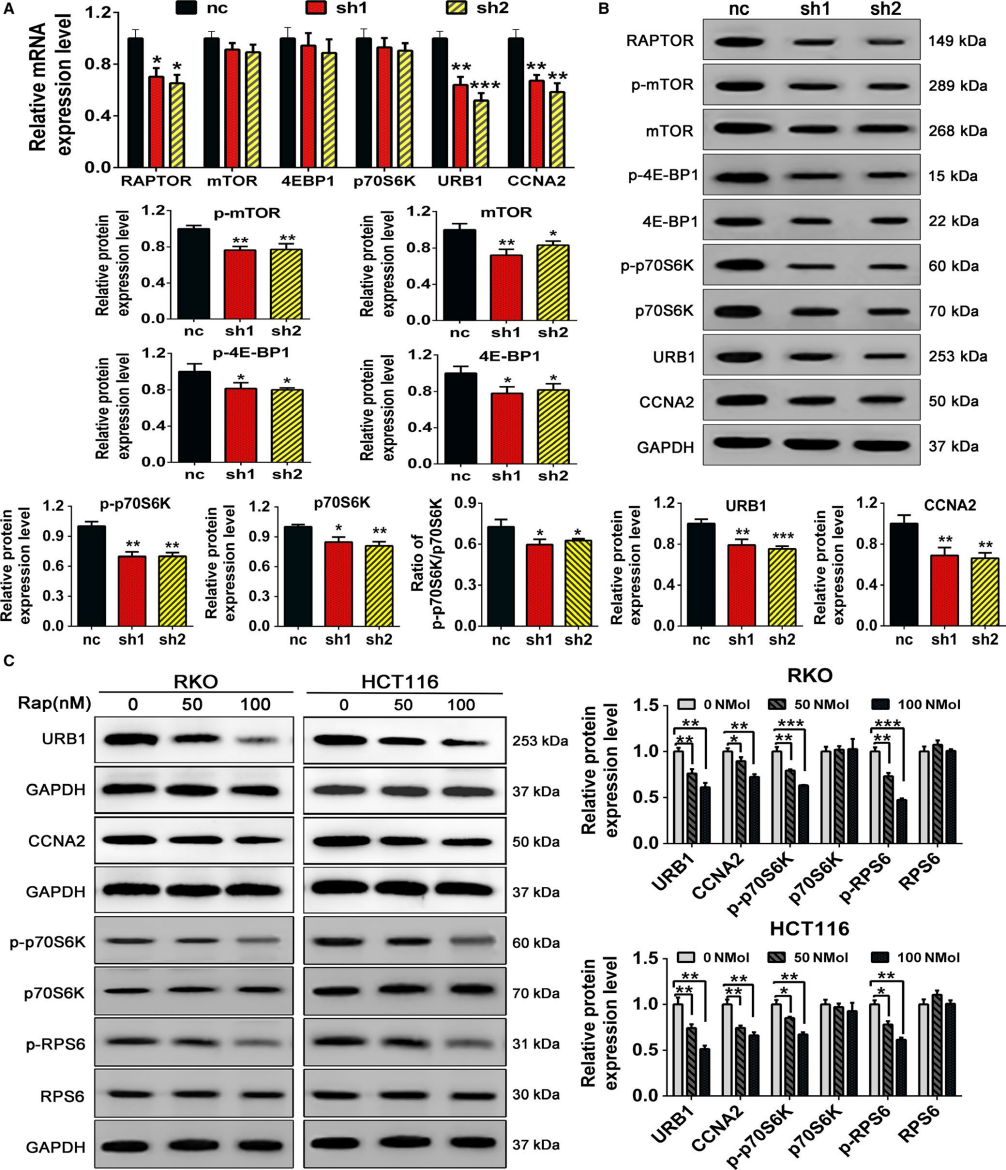
Regulatory associated protein with mammalian/mechanistic target of rapamycin (RAPTOR) silencing or rapamycin treatment inactivates mTOR complex 1 (mTORC1) and downregulates URB1 and cyclinA2 (CCNA2) expression. A and B, The mRNA and protein expression levels of mTORC1 components and substrates. The mRNA and protein levels of URB1 and CCNA2 in RAPTOR silencing RKO cells were measured via quantitative real‐time PCR and western blot, respectively. C, Western blot was used to assess the inhibitory effect of rapamycin at different concentrations on URB1 and CCNA2 expression and mTORC1 activity in CRC cells. Rapamycin concentrations used in this study included 50 and 100 nmol/L. Data are mean ± SD (n = 3), *P < .05, **P < .01 and ***P < 0.001

To confirm whether RAPTOR regulated mTORC1 signaling activity in CRC cells, we examined the influence of RAPTOR silencing on mTORC1 key components and phosphorylated substrates. The mRNA expressions of *mTOR*, *p70S6K,* and *4EBP1* were not significantly influenced by the loss of RAPTOR in RKO cells (Figure 4A), which implied that RAPTOR may not regulate mTORC1 signaling at the transcriptional level. We further detected the phosphorylation of RAPTOR on mTORC1 key components and substrates by Western blotting analysis. Indeed, RAPTOR silencing dramatically decreased the protein level of key components and substrates of mTORC1 (Figure 4B). The regulation of RAPTOR on URB1 and CCNA2 were also measured, and interestingly, both the mRNA and protein level of URB1 and CCNA2 were downregulated by RAPTOR silencing (Figure 4A,B). Furthermore, rapamycin, a specific inhibitor of mTORC1, was used to validate the activation effect of mTORC1 signaling on URB1 and CCNA2. Our results showed that rapamycin synchronously inhibited the protein expression of URB1, CCNA2, p‐p70S6K, and p‐RPS6 in a concentration‐dependent manner (Figure 4C). Taken together, these results further supported that RAPTOR might activate URB1 and CCNA2 via the mTORC1 signaling pathway.

### 3.5 Ectopic overexpression of RAPTOR promotes the proliferation and cell cycle progression of CRC cells via activation of mTOR and upregulation of URB1 and CCNA2

**Figure 5:**
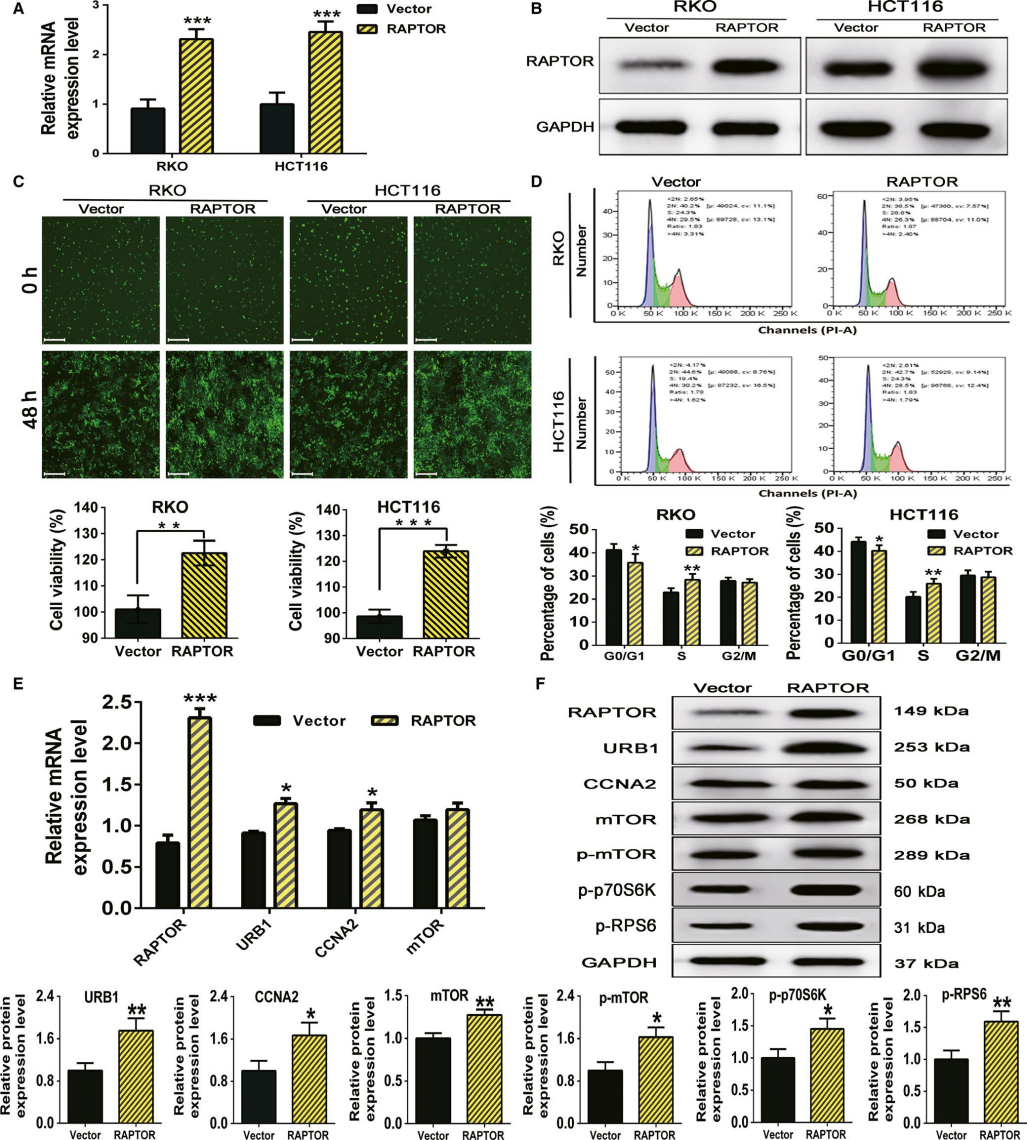
Ectopic overexpression of regulatory associated protein with mammalian/mechanistic target of rapamycin (RAPTOR) promotes the proliferation and cell cycle progression in colorectal cancer (CRC) cells by activating mTOR complex 1 and upregulating URB1 and cyclinA2 (CCNA2) expression. A and B, The transfection efficiency of RAPTOR overexpression plasmids in CRC cells was measured by using quantitative real‐time PCR (qRT‐PCR) and western blot. RAPTOR represents the RAPTOR overexpression group, and Vector represents the control group. C, Cell viability was determined by a CCK‐8 assay after RKO and HCT116 cells were transfected with RAPTOR overexpression and negative control vector plasmids. Scale bars: 50 μm (×100 magnification). D, The images show that the cell cycle progression of CRC cells was acutely promoted by RAPTOR overexpression. E and F, By using qRT‐PCR and western blot analysis, overexpression of RAPTOR distinctly provoked mTORC1 signaling and increased URB1, and CCNA2 expression in RKO cells. Data are mean ± SD (n = 3), *P < .05, **P < .01 and ***P < .001

The RAPTOR overexpression plasmid (named RAPTOR) or control plasmid (named Vector) were transfected into RKO and HCT116 cells, and the transfection efficiency was examined by qRT‐PCR and western blotting. The results indicated that endogenous expression of RAPTOR in CRC cells was significantly elevated upon transfection of the RAPTOR plasmid as compared to transfection of the vector plasmid (Figure 5A,B). Cell Counting Kit‐8 and FCM assays demonstrated that overexpression of RAPTOR promoted the proliferation and cell cycle progression of CRC cells (Figure 5C,D). In addition, using qRT‐PCR, we uncovered that the mRNA levels of *URB1* and *CCNA2*, but not *mTOR* were prominently upregulated by RAPTOR overexpression (Figure 5E). Furthermore, the protein expression level of URB1, CCNA2, mTOR, p‐mTOR, p‐p70S6K, and p‐RPS6 were all markedly increased by RAPTOR overexpression, as shown by Western blotting (Figure 5F). In brief, these data indicated that RAPTOR might play an oncogenic role in the proliferation and cell cycle progression of CRC cells via activating mTORC1 and upregulating URB1 and CCNA2.

### 3.6 RAPTOR contributes to human CRC xenograft proliferation by provoking mTORC1 and upregulating URB1 in vivo

**Figure 6:**
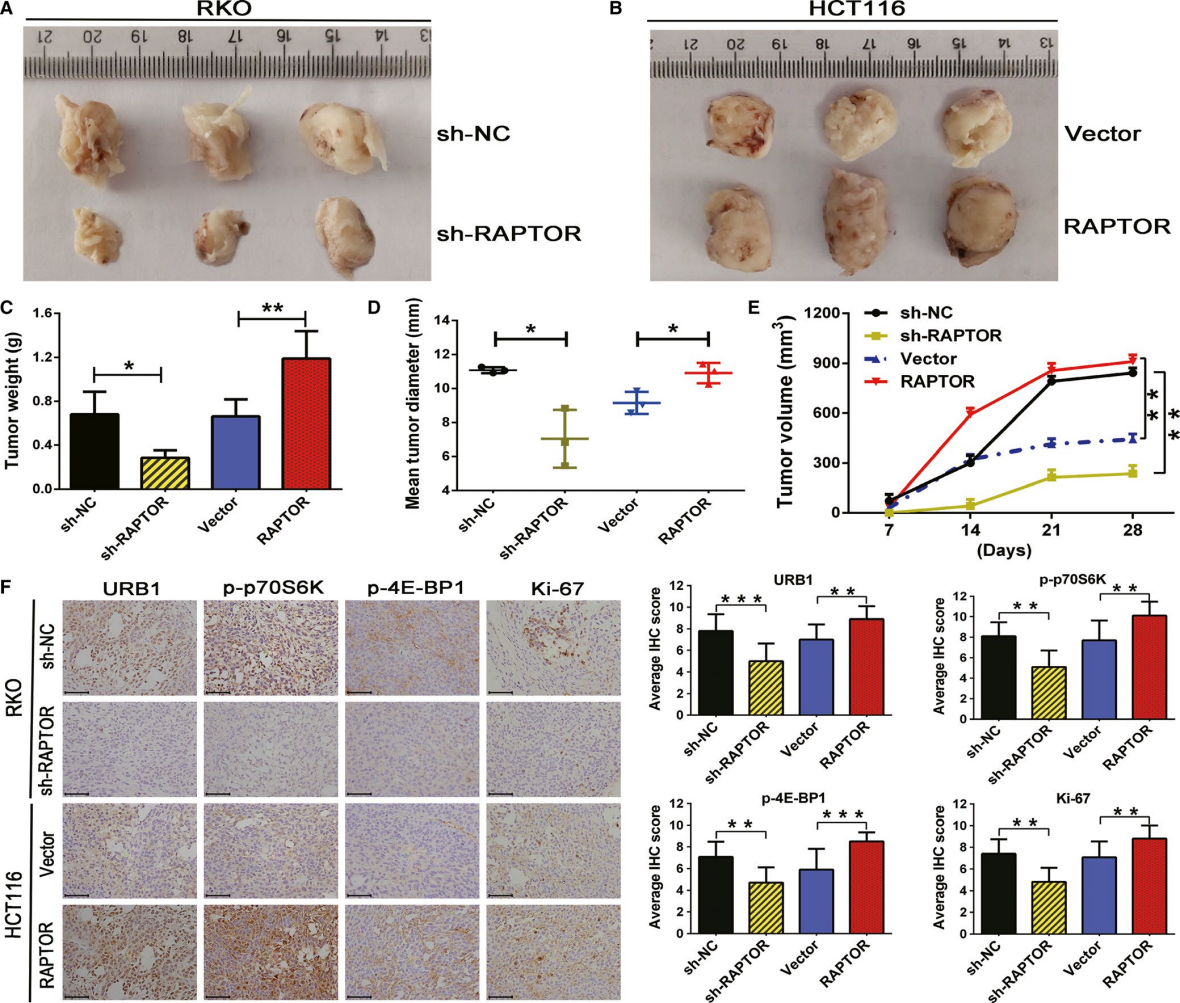
Oncogenic functions of regulatory associated protein with mammalian/mechanistic target of rapamycin (RAPTOR) on colorectal cancer (CRC) cells in vivo. RKO cells were transfected with sh‐RAPTOR (n = 5) or ctrl shRNA (sh‐NC) lentivirus (n = 5) (A), and HCT116 cells were transfected with negative control Vector (n = 5) or RAPTOR (n = 5)overexpression plasmids (B), and then both cells were injected subcutaneously into nude mice. C‐E, Tumor weight, tumor diameter, and tumor volume were monitored to calculate the differences of tumor growth between the indicated groups (n = 5). F, Immunohistochemistry (IHC) staining was used to detect the regulatory effect of RAPTOR on mTORC1 activity and URB1 expression in vivo. Scale bars: 50 μm (×400 magnification). Data are mean ± SD (n = 5), *P < .05, **P < .01 and ***P < .001

**Figure 7:**
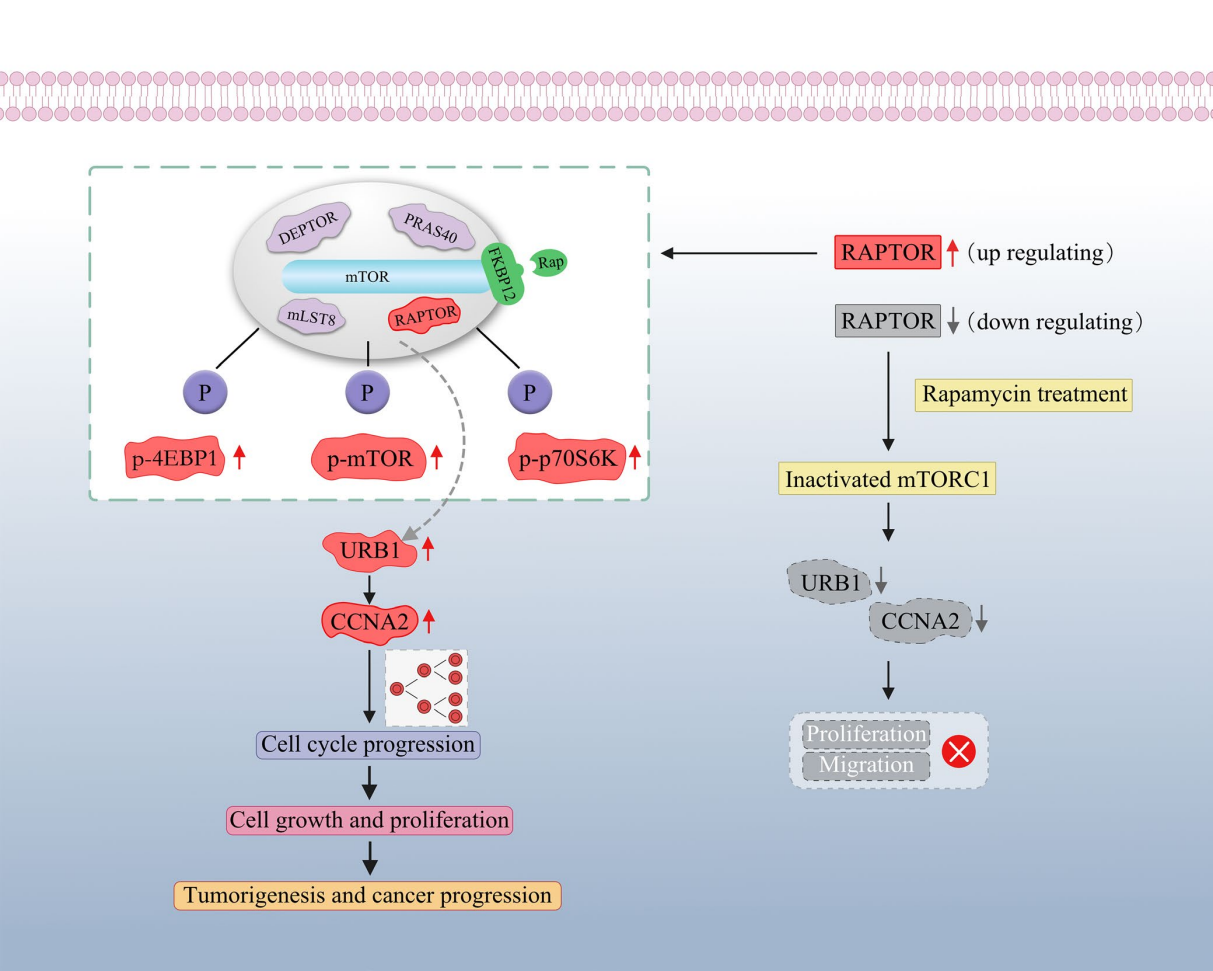
Schematic diagram showing the effect of regulatory associated protein with mammalian/mechanistic target of rapamycin (RAPTOR) on tumorigenesis and progression of colorectal cancer (CRC) by targeting URB1. Upregulation of RAPTOR promotes proliferation and cell cycle progression of CRC cells by inducing mTORC1 signaling and upregulating URB1 and cyclinA2 (CCNA2) expression. RAPTOR silencing or rapamycin treatment inactivates mTOR complex 1 and impairs URB1 and CCNA2 transcription, which blocks the proliferation and cell cycle transition and induces apoptosis of CRC cells

Since RAPTOR promoted CRC cell proliferation and activated mTORC1 in vitro, we next sought to investigate whether RAPTOR exerted the same oncogenic function in vivo. Thus, RAPTOR knockdown in RKO cells (named sh‐RAPTOR group), RAPTOR overexpression in HCT116 cells (named RAPTOR group), and the respective control groups (named sh‐NC and Vector group, respectively) were injected subcutaneously into nude mice in order to explore the precise role of RAPTOR in vivo. As expected, the growth of xenografts in the sh‐RAPTOR group was significantly slower than sh‐NC groups, as evidenced by the tumor weights and diameters. On the contrary, xenografts of the RAPTOR group exhibited a faster growth, larger size, and heavier tumor in comparison to the Vector group (Figure 6A‐E). Furthermore, in order to investigate the role of RAPTOR in activating mTORC1 and regulating URB1 in vivo, IHC was used to measure the protein expression of URB1 and mTORC1 canonical phosphorylation substrates. According to the IHC staining, we found that the protein expression level of p‐4EBP1, p‐p70S6K, URB1, and cell proliferation marker Ki‐67 were all lower in the sh‐RAPTOR group than in the sh‐NC group. Moreover, these proteins exhibited a higher expression level in the RAPTOR group as compared to those of the vector group (Figure 6F). Thus, we concluded that RAPTOR promoted the proliferation and growth of CRC cells by activating mTORC1 and transcriptionally upregulating the ribosomal assembly factor, URB1 (Figure 7).

## 4 DISCUSSION

In this study, we demonstrated that the mTORC1 key component RAPTOR and ribosome assembly factor URB1 were synchronously upregulated and had a positive correlation expression pattern in CRC. High expression of RAPTOR is indicative of more infaust clinicopathological features and poorer prognosis in comparison to a low expression group. Moreover, RAPTOR silencing significantly inhibited proliferation and migration and induced apoptosis in CRC cells, in addition to markedly inactivating mTORC1 and downregulating URB1 expression. Additionally, ectopic overexpression of RAPTOR resulted in a reverse effect as mentioned above. In addition, by utilizing the mTORC1 specific inhibitor rapamycin, we further confirmed that URB1 was dramatically regulated by mTORC1 signaling. Together, these findings indicated that RAPTOR promotes CRC tumorigenesis and progression by provoking mTORC1 and the transcriptional activation of URB1, which further supports the potential oncogenic role of mTORC1/RAPTOR‐URB1 axis in neoplasia.

RAPTOR, a 150 kDa mTOR‐binding protein, is localized on chromosome 17q25 and, conserved within various eukaryotes, and is responsible for the excitation of mTORC1.^23^, ^24^ mTORC1/RAPTOR signaling regulates various biological functions—including ribosomal biogenesis, energy metabolism, and autophagy—and is involved in organ development, aging, and tumorigenesis. Knockdown of RAPTOR in mice induces spermatogonia self‐renewing disorder, which indicates the cell autonomous requirement for RAPTOR in the formation and maintenance of the spermatogonial stem cell pool and underscores the importance of mTORC1 in male germ cell development.^25^ Hepatoid adenocarcinoma of stomach (HAS) is characterized by histological resemblance to HCC and has a poor prognosis. It has been reported that high‐frequency mutations of RAPTOR (13%) and TP53 (30%) are intimately associated with HAS malignant transformation and progression.^26^ Moreover, subcellular localization of RAPTOR might admonish an idiosyncratic phenotype of tumor cells. Accumulation of nuclear RAPTOR has been demonstrated to promote ER‐phosphorylation and mediate tamoxifen resistance, and predicts poor prognosis of ER‐positive breast cancer.^27^ These findings strongly argue that dysregulation of RAPTOR contributes to neoplasia and malignancy. Consistent with the studies above, we demonstrated that RAPTOR was overexpressed at both the mRNA and protein level in CRC tissues, and high expression levels of RAPTOR predicted more vicious clinical characteristics and poorer OS (Figure 1). A previous study reported that as a autophagy‐related gene, RAPTOR is related to the progression and multidrug resistance in CRC,^28^ which further supports our standpoint. Additionally, we uncovered that RAPTOR silencing prominently impeded the proliferation and simultaneously induced cell cycle arrest and apoptosis in CRC cells in vitro. In summary, our results demonstrated that RAPTOR acts as a canonical oncogene in CRC initiation and development.

In order to confirm the underlying correlation of oncogenic function and RAPTOR‐dependent activation of mTORC1, we performed a combination of pharmacological and genetic approaches to assess the activity of total and phosphorylated mTORC1 activity in CRC. As shown in Figure 4, mTORC1 activity was quenched by RAPTOR knockdown, while overexpression of RAPTOR performed a completely reverse effect (Figure 5). We also uncovered that mTOR protein, rather than mTOR mRNA, was influenced by RAPTOR manipulating, indicating that RAPTOR may regulate mTOR via translational or posttranslational modifications, at least in part by phosphorylation regulation, but not in a transcriptional manner. More interestingly, p‐p70S6K expression was dramatically decreased by RAPTOR silencing as compared to p‐4EBP1 (Figure 4B). This result was similar to results of previous studies, which illustrated that rapamycin was a partial mTORC1 inhibitor and induced the inhibition of p‐p70S6K/ RPS6, but with limited effects on p‐4EBP1/eIF4E axis.^29^, ^30^ Coincidently, there have been a larger number of investigations demonstrating the association of RAPTOR and mTORC1 activity in various cancers. Activation of the mTORC1 cascade was essential for c‐Myc‐dependent hepatocarcinogenesis. Ablation of RAPTOR strongly inhibited c‐Myc liver tumor formation via quenching mTORC1 cascades and downregulating SLC1A5 and SLC7A6, which further weakened mTORC1.^7^ LncRNA MALAT1 contributed to HCC development by enhancing the translation of the metabolic transcription factor TCF7L2. Pharmacological or genetic inhibition of mTOR and RAPTOR or expression of a hypophosphorylated 4EBP1 resulted in decreased expression of TCF7L2, which relieved the oncogenic effect of MALAT1.^31^ NPRL2 is a newly identified tumor suppressor and is located in the lysosomal membranes. A previous study showed that NPRL2 interacts with RAPTOR to activate mTORC1 in an amino acid‐dependent manner,^32^ indicating the involvement of amino acid metabolism in regulating mTORC1/RAPTOR signaling. However, it was recently reported that mTORC1 activation may suppress tumorigenesis in patients with inflammatory bowel disease who develop CRC via chromosomal aberrations and IL‐6‐associated inflammation blocking, but may promote CRC oncogenesis in patients with APC mutations, which demonstrates the oncogenic and tumor‐suppressive roles of mTORC1 in tumorigenesis.^33^ Thus, mTORC1 may execute completely different functions in tumors with a different pathogenesis. Although multiple and complex regulatory mechanisms coexist, our results suggest that RAPTOR benefits tumorigenic transformation and progression via provoking mTORC1 in CRC.

It has been affirmed that mTORC1 controls multiple steps of ribosome biogenesis.^15^ However, whether mTORC1/RAPTOR signaling regulates oncogenic function through transcriptional activation of ribosome assembly factors has not been elucidated. PNO1, a ribosome assembly factor, has been reported to contribute to CRC progression by negatively regulating the p53 signaling pathway.^17^ This study encouraged us to explore the latent relationship between mTORC1/RAPTOR signaling and ribosome assembly factors in tumorigenesis. URB1, also known as ribosome biogenesis homolog 1, is a classical ribosome assembly factor and accounts for the early steps of 60S ribosomal subunit biogenesis.^34^ In our ongoing investigation, we have confirmed that URB1 was overexpressed in CRC and promoted CRC proliferation and upregulation of CCNA2. It is well known that CCNA2 is a downstream target of the tumor suppressive signaling p53/p21 pathway and participates in cell cycle transition.^35^, ^36^ Overexpression of CCNA2 in PDAC predicts a more infaust histological grade, worse OS, and more advanced tumor stage.^37^ CCNA2 was shown to act as a oncogene and played a crucial role in regulating cancer cell growth and apoptosis, serving as a new biomarker for the diagnosis and therapy of CRC.^38^, ^39^ These findings support the potential of URB1 and CCNA2 as a linkage of crosstalk between mTORC1 and p53/p21 signaling. Thus, it is reasonable to propose that the essential mTORC1/RAPTOR‐URB1‐CCNA2 axis plays a role in tumorigenesis.

By using IHC staining, we identified that URB1 expression was positively related to RAPTOR. Furthermore, through a combination of pharmacological and genetic approaches, we determined that mTORC1/RAPTOR signaling distinctly controlled the mRNA and protein expression of URB1 and CCNA2. Our previous study revealed that URB1 regulated CCNA2, and CCNA2 has been shown to control cell cycle transition. Thus, it is confident to suggest that mTORC1/RAPTOR signaling might promote cell cycle progression and proliferation in CRC cells via transcriptional activation of URB1 and CCNA2. Furthermore, silencing of RAPTOR induced cell cycle arrest and apoptosis, which might have resulted from URB1‐mediated CCNA2 downregulation.

In conclusion, our study found that RAPTOR and URB1 were significantly overexpressed in CRC and had a positive correlation. RAPTOR silencing inhibited proliferation and induced cell cycle arrest, and apoptosis of CRC cells by inactivating mTORC1 and suppressing URB1 and CCNA2 transcription. Our findings suggested that RAPTOR serves as a favorable target to block mTORC1‐ribosome assembly factors signaling cascade. Furthermore, inhibition of RAPTOR might be a promising strategy for the treatment of CRC.

## CONFLICT OF INTEREST

None declared.
