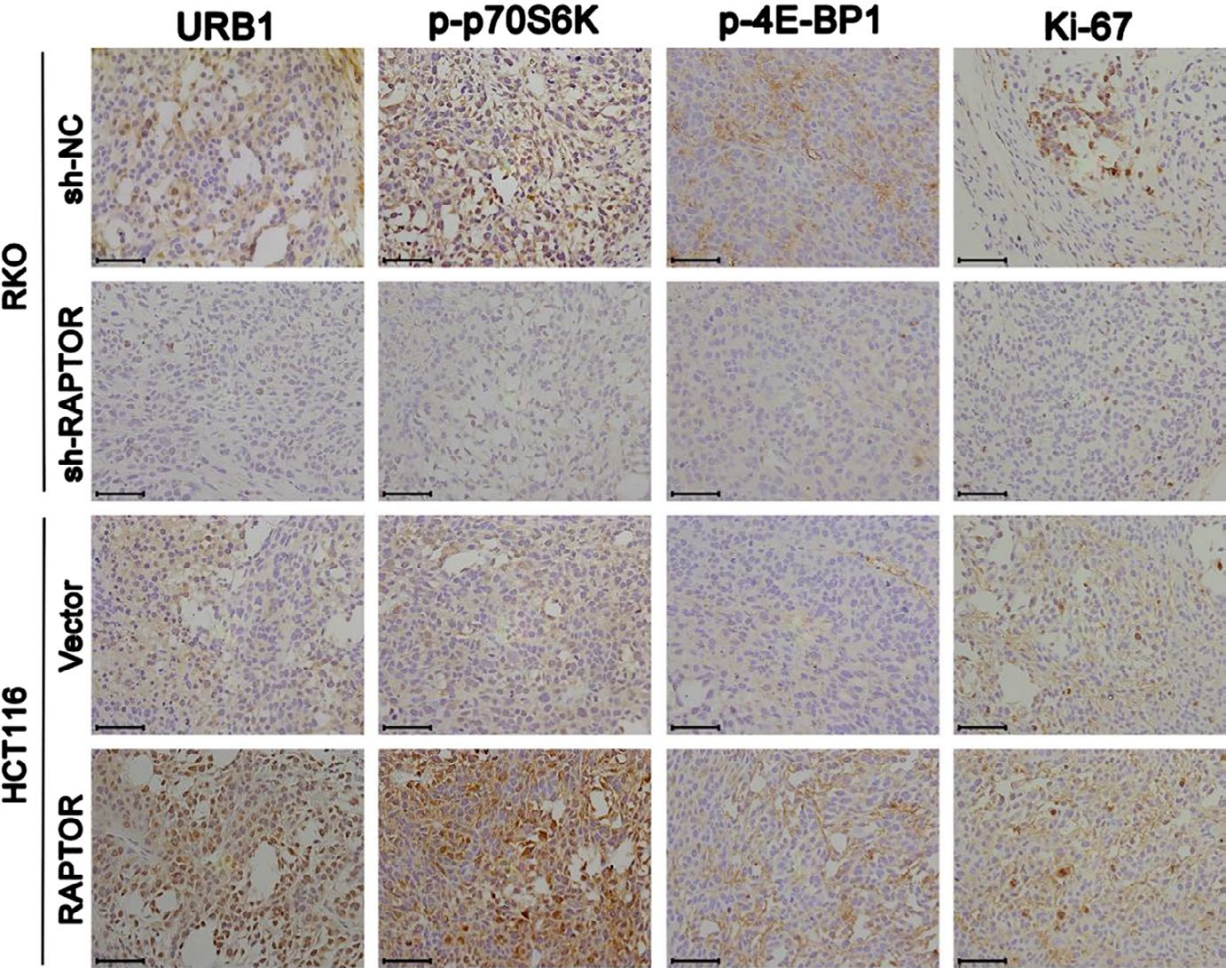# Corrigendum

**DOI:** 10.1002/cam4.4337

**Published:** 2021-10-04

In the article by Wang et al.,^1^ entitled “RAPTOR promotes colorectal cancer proliferation by inducing mTORC1 and upregulating ribosome assembly factor URB1,” the author wants to correct figure parts 2F, 3B, and 6F.

Corrected Figure 2F

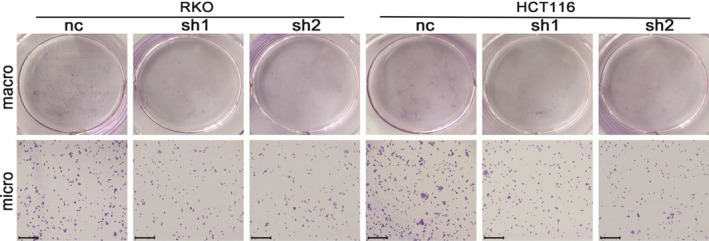

Corrected Figure 3B

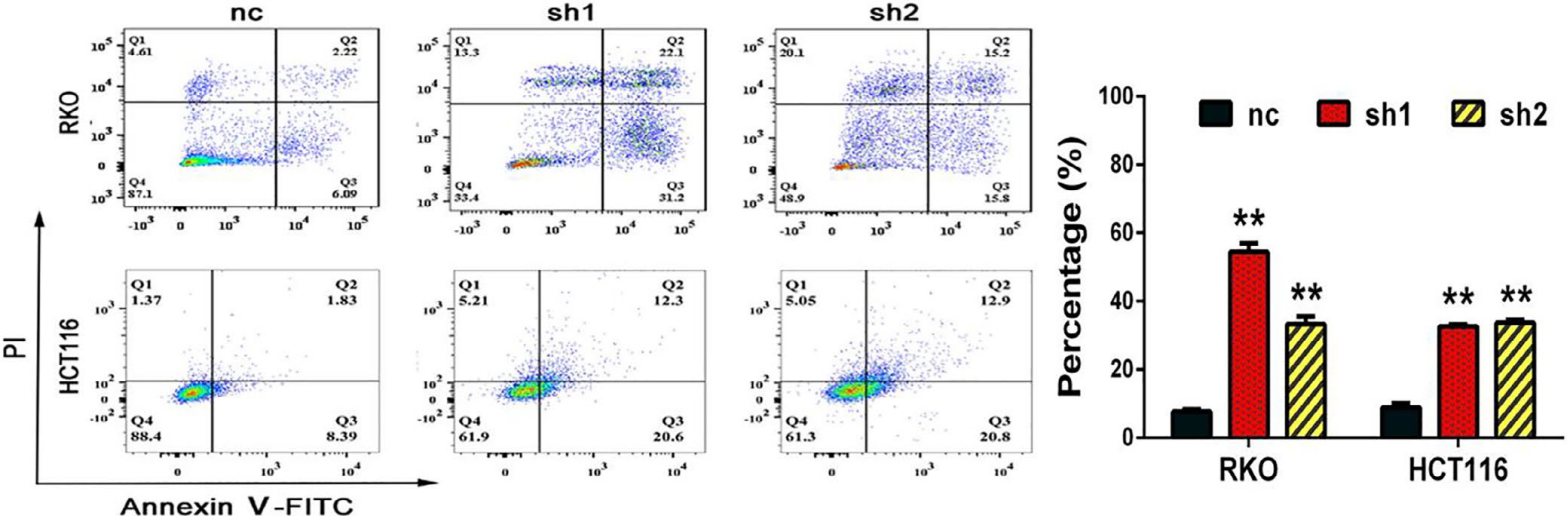

Corrected Figure 6F